# Patient and nurse perspectives of a nurse-led community-based model of HIV care delivery in Malawi: a qualitative study

**DOI:** 10.1186/s12889-020-08721-6

**Published:** 2020-05-14

**Authors:** Odala Sande, Doris Burtscher, Daneck Kathumba, Hannock Tweya, Sam Phiri, Salem Gugsa

**Affiliations:** 1 Lighthouse Trust, Lilongwe, Malawi; 2 Médecins Sans Frontières, Vienna Evaluation Unit, Vienna, Austria; 3 University of Washington, Seattle, WA USA

**Keywords:** HIV, ART, Differentiated models of care, Community-based, Retention, Malawi

## Abstract

**Background:**

Differentiated models of care (DMOC) are used to make antiretroviral therapy (ART) accessible to people living with HIV (PLHIV). In Malawi, Lighthouse Trust has piloted various DMOCs aimed at providing quality care while reducing personal and logistical barriers when accessing clinic-based healthcare. One of the approaches was community-based provision of ART by nurses to stable patients.

**Methods:**

To explore how the nurse-led community ART programme (NCAP) is perceived, we interviewed eighteen purposively selected patients receiving ART through NCAP and the four nurses providing the community-based health care. Information obtained from them was complemented with observations by the study team. Interviews were recorded and transcribed. Data was analysed using manual coding and thematic analysis.

**Results:**

Through the NCAP, patients were able to save money on transportation and the time it took them to travel to a health facility. Caseloads and waiting times were also reduced, which made patients more comfortable and gave nurses the time to conduct thorough consultations. Closer relationships were built between patients and care providers, creating a space for more open conversations (although this required care providers to set clear boundaries and stick to schedule).

Patients’ nutritional needs and concerns related to stigma remain a concern, while operational issues affect the quality of the services provided in the community. Considerations for community-led healthcare programmes include the provision of transportation for care providers; the physical structure of community sites (in regard to private spaces); the timely consolidation of data collected in the field to a central database; and the need for care providers to cover multiple facility-based staff roles.

**Conclusions:**

The patients interviewed in this study preferred the NCAP approach to the facility-based model of care because it saved them money on transport, reduced waiting-times, and allowed for a more thorough consultation, while continuing to provide quality HIV care. However, when considering a community-level DMOC approach, certain factors – including staff transportation and workload – must be taken into consideration and purposefully planned.

## Background

In Malawi, the prevalence of HIV among persons aged between 15 and 64 is 10.6% [1]. The country started providing antiretroviral therapy (ART) in public hospitals in 2003. Since then, ART delivery has been expanded through decentralising services from hospitals to peripheral health centres. In 2016, in line with WHO guidelines recommending that all people living with HIV should be provided with ART regardless of their CD4 count [2], Malawi adopted the ‘test and treat’ strategy [3].

As the number of people on ART in low-income countries increases, the challenges of delivering lifelong treatment become harder to tackle and require innovative solutions to ensure efficient services, without compromising on quality. Given that approximately one third of patients initiated on ART are documented as lost to follow-up (LTFU) within 2 years [4, 5], national HIV programmes are building strategies to ensure that more patients remain in care [6–8].

Studies on attrition rates have found that patients are most likely to default from care during the initial 12 months of treatment, mainly due to high mortality rates [9]. Loss to follow up was identified as the main reason for attrition after 12 months of treatment. In recent years, differentiated models of care (DMOCs) have been implemented to provide HIV care and treatment services tailored to patients’ needs, including the type of visit (drug refill only, or inclusion of thorough clinical assessment during the visit) and location of service. In some models of care, services are dedicated and tailored to specific groups such as teenagers and pregnant women. In others, frequency of clinical assessments (every 1-, 3- or 6-months) and also adherence support activities have been prioritised based on patients’ needs.

Several implementation studies, involving stable patients with sustained adherence on ART in Mozambique and Malawi, have tested ART distribution models and adherence monitoring that is carried out by patients themselves [10–12]. These approaches separate the need for ART delivery from the need for clinical assessment [13]. By doing so, such community-based approaches addressed the practical barriers patients face when accessing ART, and also contributed to improved peer support, a factor patients considered fundamental to their wellbeing [14–16]. Despite its alleviation of some patient burdens, the approach was not well-received by ministries of health due to the risks of such complex care being delivered by people with no medical experience. In order to achieve a patient-centred approach which was also accepted by local health authorities, Lighthouse Trust implemented a modified version in Malawi using community-based nurses as care providers in 2016. This nurse-led community ART programme (NCAP) was developed specifically for patients who were adherent on ART.

Most of the previous studies on DMOCs have focused on patient experience and perception [4, 6, 14, 15, 17–21], and few have included the perceptions of the service providers [22]. This study used qualitative methods to explore both nurses’ and patients’ perceptions of providing and receiving HIV care in a nurse-led community-based model during the two-year implementation of the programme.

## Methods

### Study design

This study used qualitative methods to explore the perceptions and experiences of NCAP nurses and patients, including benefits and challenges of providing and receiving a community-based ART service [23]. After purposive sampling of participants, data was collected through in-depth interviews with key participants and observation of sessions. A methodological triangulation of findings was undertaken to enhance the interpretation of data. In-depth individual interviews were combined with focus group discussions, observations and a literature review. This enabled an accurate representation through use of multiple methods or perspectives [24].

### Study setting

The study was conducted in urban and peri-urban parts of Malawi’s capital Lilongwe, where NCAP was implemented by Lighthouse Trust. As of December 2017, Lighthouse Trust was one of the largest providers of ART in Malawi’s central region with over 28,000 patients currently on ART at the KCH and MPC alone. By the end of November 2018, after 2 years of implementation, 1366 patients were accessed care through NCAP. These 1366 patients belong to 41 peer support groups registered with Lighthouse Trust. Data on routine services provided to patients is first captured on paper-based patient charts and later entered in an electronic monitoring record system that is routinely used at the two clinics. The services provided in the community are the same as those provided at the fixed clinics, except where referral for specialised care is necessary.

### Study population

NCAP was offered to registered patients at two hospitals with a high caseload: Lighthouse Clinic at Kamuzu Central Hospital (KCH) and Martin Preuss Centre (MPC) at Bwaila Hospital, both in Lilongwe, Malawi. The inclusion criteria for care is being (1) an adult (i.e. aged 18+ years); (2) on the same first or second line ART regimen for at least 6 months; (3) who had been declared ‘stable’ by a healthcare provider; (4) who had no serious side effects or opportunistic infections; (5) were virally suppressed or undetectable; (6) lived within Lighthouse Community Health Services (CHS) catchment areas; and (7) were a member of peer support group. Under NCAP, eligible patients can access care in their communities instead of travelling to a fixed facility. Patients collaborate with the CHS team to identify the place where they can receive care and collect antiretroviral drugs (ARVs) during their appointment dates. Health promoters follow up with those who miss their appointments and provide feedback to the nurses.

The participants in this study are patients enrolled in the NCAP and the nurses who provide the service in the patients’ communities. A purposive sampling method was used to recruit participants because of accessibility to and homogeneity of the target population. In-depth interviews were conducted with 18 patients from five selected support groups located in semi-urban and rural parts of Lilongwe, until a point of saturation was reached on themes being explored. In-depth interviews were conducted with all of the four community nurses.

### Data collection

Data collection took place from July to September in 2018. Informed verbal consent was obtained from participants. Interviews were conducted at community sites on the days when NCAP providers had appointments with patients. Sessions were observed to assess the interactions between nurses and patients at the NCAP outreach sites, and compared with what was described during interviews [25, 26]. Observations took place on 3 days for a minimum of one hour. Patients-to-nurse interaction, time spent with the nurses and how patients interacted with each other were observed and recorded.

In-depth interviews were conducted using a topic guide (Additional file 1 & 2) to explore an emic perspective [27]. Interviews with patients focused on their perception of the services provided through NCAP, including the perceived benefits and challenges of providing and receiving care in the model. The lead author visited the sites where the support group members met on their appointment dates and asked participants for an interview. Interviews were held in a discrete place, conducted by a native Chichewa speaker and audio recorded. Audio files were translated and transcribed in English. Interview transcripts were supplemented with handwritten field notes.

Interviews with four nurses were conducted in a private room in fixed clinics. Nurses were also asked what they thought about the model, including what they perceived to be the benefits and the challenges of delivering services using this model.

### Data analysis

The interview transcripts did not include any personal identifiers to protect the confidentiality of the participants. Data analysis was conducted manually by one researcher and then validated by two senior researchers. This involved thematic content analysis; transcriptions were screened for relevant information which was then organised, coded, categorised and interpreted. A code was attached to statements from the transcripts in order to structure the data [28]. The content was analysed in two ways: by describing data without reading anything into it, and interpretatively by focusing on what was meant by the responses [29]. The empirical data was analysed in an inductive way, in which codes were generated based on the data that was gathered. From these codes, main themes were extracted.

Continuous reflection on data was part of the creative process of analysis and necessary for contextualising and linking findings with theory. Validation of data analysis occurred through methodological triangulation [24].

## Results

Three main themes emerged from the interviews: 1) The perceptions of both patients and nurses of the community-based care model, 2) closer relationship between patients and care providers and 2) operational challenges related to community-based care. Logistical benefits for patients and barriers to sustained self-care emerged as subthemes of patients’ and nurses’ perceptions. Management of transport heath care providers, punctuality of patients, data collected in the field, space for discussing private issues emerged as subthemes of operationalizing a community ART delivery services.

Patients and nurses had different perceptions and experiences of the NCAP. While patients reflected mainly on the positive experiences of receiving care through NCAP, nurses described both the positive and challenging aspects of the programme. The main themes that emerged were logistical benefits to patients, barriers to sustained self-care, closer relationships between patients and care providers, and operational challenges faced by nurses when delivering care.

Fourteen female and four male patients were interviewed (see Table 1). The age of support group members interviewed ranged from 28 to 61. Additionally, one female and three male nurses, with one to 3 years’ experience running NCAP, participated in the interviews.

**Table 1:** Characteristics of stable patients interviewed

Demographic information	Number
Total	18
Gender	
Female	14
Male	4
Age range	
26–35	3
36–45	10
46–55	2
56–65	3
Geographical location	
Semi-urban	9
Rural Area	9

### Patient and nurse perceptions

Nurses and patients reported that the NCAP helped patients save time and money. Participants also felt positive about the personal interaction between patients and nurses; with patients feeling comfortable and well treated, and nurses able to play greater role in caring for patients. However, despite the money saved on transport, reduced commuting and clinical waiting times, and ‘comfortable’ environment for receiving care, patients continued to face financial limitations and stigma in their communities.

*Money saved on transport:* Most of the patients reported saving the money they would have spent on public transport and using it for other purposes. This was also echoed by nurses:

*It is better to be getting the drugs in the community because we do not spend money on [public] transport, money is saved as the drugs are brought to us, which to me is a good thing. (female patient; age range 26–35)*

*I was very happy especially considering transport. I considered it to be very important, nothing can beat bringing the drugs to the community. (male patient; age range 56–65)*

*It costs [the patients] K1000 [1.40 USD] just for [public] transport, excluding other expenses. When [the patients] are home they can use that K1000 to buy relish [food] and prepare food which can be helpful for their health. (Nurse 3)*

*Reduced commuting and clinic waiting times:* NCAP participants described a significant reduction in the time it took them to get to a clinic and be seen by a healthcare provider.

*We used to wait a long time at the clinic. There were a lot of people. Here in the community we collect the drugs once the nurse arrives and I am done for the day. […*] *I don’t lose anything like transport money or time. (female patient; age range 36–45)*

Patients reported that the time they saved allowed them to work, care for their families, or get more involved in community activities.

*It gives me more time to go and order things for my business. I will continue selling when I am done here. So, my business is not suffering. (female patient; age range 56–65)*

*It helps me to look after the children. I can take care of them. (female patient; age range 36–45)*

*The activities in their community that required their presence made it difficult for them to report to the clinic, but through this programme [NCAP] they know that they can come to us and we will assist them, and they then can participate in community activities, such as funerals. (Nurse 1)*

*‘Comfortable’ environment:* Nurses and patients described the community-based locations to be comfortable because they were less crowded than the fixed clinics.

*I can see that it goes well, and I will be leaving in good time. There is nothing like struggling and waiting in a queue at the clinic. (female patient; age range 36–45)*

*The most important thing I observe is that people are comfortable. Some people tend to be uncomfortable once they see the clinical setting. (Nurse 2)*

Patients were less stressed when planning a clinic visit through the NCAP, because they didn’t have to make additional arrangements for a longer time away from home and work. With NCAP, patients didn’t have to drastically change their routine – they started their day at a normal hour and were able to access care in a more relaxed way.

*I was impressed with the outline [of the programme] because I used to be worried when my appointment date approached. (female patient; age range 46–55)*

*We were worried at the time that we were getting [ARVs] from Lighthouse. We had to think that “tomorrow I will have to go to the clinic to get the drugs”. (female patient; age range 46–55)*

*Lack of money to buy food:* Even though some participants used the money they saved to buy food, both nurses and patients mentioned that lack of food remains a significant challenge for patients.

*Sometimes I come here while hungry, I do not eat because of lack of food (female patient; age range 36–45)*

*Something that is not available [at the NCAP] is food. (female patient; age range 36–45)*

*There are a lot of things that those who are HIV positive face. It is not only drug related. There are problems regarding nutrition. For one to be healthy, it is not only ART that is needed but also nutrition. People need money to find food. (Nurse 3)*

*Perceived stigma:* Some patients discussed about their friends who were afraid of being stigmatised by the community if they received care in their community (near their homes), and still preferred to go to clinics that were away from their homes, so they wouldn’t be recognized as a person infected with HIV by their neighbours and friends.

*My friends who are ashamed to come here, I told them that you are ashamed but that does not help you. They don’t want to show themselves to other people with fear that they might be laughed at. (male patient; age range 26–35)*

*A lot of people still go to the clinic saying they can’t get the drugs on an open place like this one. We accepted that we have HIV. This is our life and even if we hide, who will we be hiding from? (female patient; age range 36–45)*

However, one nurse reflected that the NCAP had the potential to combat community stigma by demonstrating that HIV care is not something to hide and can be successfully treated outside of a fixed clinic setting.

### Closer relationship between patients and care providers

Participants noted improved interaction between the nurses and patients because there are fewer patients than in the fixed clinics, and nurses can therefore dedicate more ‘quality’ time to every consultation.

*As well as ARV refill, we also provide drugs for other health-related problems that patients report. We refer them to the clinic if there are problems that need to be seen by a doctor. (Nurse 2)*

*They took care of us just as they would at Lighthouse. They are respectful and have humour. They also give a person a chance to say what they want. They document in a proper manner unlike at the clinic where they might be fast and give you back your health passport before you even finish explaining. (male patient; age range 56–65)*

Compared to clinic-based visits where care providers see hundreds of patients daily, those receiving care from NCAP had more time during consultations to report other illnesses they might be suffering from. Nurses continued to document the outcomes of consultations in the patients’ health card.

Some nurses felt that although the increased time with patients led to closer relationships, it was more difficult for them to establish boundaries and keep interactions professional.

*We are in one-to-one contact, [patients] tend not to be serious because there is interpersonal relationship, unlike at the clinic they meet different [health care providers]. They would not approach them the way they do in the community. (Nurse 4)*

### Operational challenges

Unlike patients, nurses described several operational challenges when running the NCAP.

*Transport:* The four nurses interviewed all experienced issues and delays with transportation which had made them late to their assigned location. Some faced similar delays when returning from the field location to the facility.

*The problem that we have is that in some cases we have challenges with transport to take us to the community because we do not have specific vehicles for the programme; this makes us late to our appointments in the community. (Nurse 1)*

*Latecomers:* Nurses reported that patients are not always on time for their appointments which adds delays to their schedule.

*Sometimes what happens is that people get used to the fact that we will go to the community. They come whenever they wish; people were coming around 1 PM, the time we actually leave, and we can’t send them back. (Nurse 2)*

*Capturing field data in a central database:* Nurses reported issues with the way data is captured and uploaded onto the central database, with delays in data entry by other facility-based staff causing confusion and inaccuracy around the follow-up status of patients.

*We write on paper during consultations and sometimes there is a delay by those who enter the information [on the system …]and this makes some of the patients to be treated as lost to follow-up. This affects us because some people lose their trust in us. (Nurse 1)*

Nurses proposed that this problem could be solved if they enter the data directly during the point-of-care, with mobile devices like tablets.

*Discussing private issues:* One nurse reported female patients were not always comfortable discussing personal, intimate issues with them. This was observed in sites where care is delivered in buildings with no private rooms.

*The patients are not comfortable to talk about certain things because of the structure [of the room]; the building is in an open space. Only in areas where they have separate rooms, patients are comfortable to report about certain things [such as sexual transmitted diseases]. That’s what I have observed and this needs to be addressed. (Nurse, 2)*

In these situations, the nurse said she would ask patients to wait for her and they would talk later behind the building.

*Covering multiple roles in the field:* Nurses shared differing views regarding the workload, but all acknowledged that their capacity was stretched when providing care in the community. The community-based nurses cover tasks usually managed by multiple facility-based staff; they take on multiple roles – receptionists, pharmacists, lab technicians, health educators, and data staff. While some nurses felt that this increased their workload, others believed it was just part of their job.

*The way we are conducting the clinic, the workload is more. As a nurse, you prepare for the clinic, you dispense the ARVs and sometimes you have to draw blood for viral load monitoring, which is a lot of tasks at once. You have to work as a pharmacist, lab technician, and you also have to go to the community to find people whom you can recruit to the programme. (Nurse 4)*

*Through [NCAP] all the tasks are done by one person, yet the same service is provided by five people at the clinic. (Nurse 1)*

## Discussion

Unlike other studies on the subject which only focus on patient experience, this study seeks to outline the perceptions of community-based healthcare providers (in this case, the four nurses providing HIV care through NCAP in Lilongwe). The findings reveal the important role that healthcare providers play in facilitating access to HIV care, benefits and challenges to the programme, and the factors to take into consideration when planning DMOC implementation.

Many of the findings from patients in this research reinforce those of previous studies: that DMOC are preferred patient initiatives for people with HIV, and help to increase accessibility to healthcare [18, 30–32]. Overall, community-based care reduced patient waiting times, transportation costs and saved them time for work and other duties at home [10, 12, 13, 33–35].

Most patients felt more comfortable accessing care through NCAP and felt less stress when planning a consultation or follow-up visit. Community sites were easier to get to which caused less disruption to their daily routines. The findings of this study confirm that community-based ART provision provides patients with the flexibility to access care closer to them [36]. The perceived benefits of NCAP are reiterated by the community-based nurses participating in this study.

While NCAP absorbed the costs for patients, it did not fully resolve issues of stigma and nutrition, which continue to pose challenges especially with retention to care [13, 17, 37–39]. Patients and nurses continue to describe HIV as a stigmatised illness which lowers people’s social capital and affects their potential to earn money to support themselves. The costs that patients save through DMOCs may not fully alleviate poverty, but it does help ease the burden of self-management: patients have more money to buy food and more time to spend with family or find work.

Contrary to patients’ concerns around stigmatisation, the nurses interviewed thought that NCAP had the potential to reduce stigma by serving as a model that integrates HIV care into the community. This approach has been proven through research in Eswatini where community-based DR-TB treatment has significantly reduced stigma [40].

Unlike other studies that explored patient-led community-based ART distribution, this study used data from a nurse-led HIV programme provided in the community. While this approach ensures the quality of the service, it is important to note that the costs saved by patients were absorbed by care providers. While transportation costs and clinic waiting times were no longer barriers for patients accessing care, nurses faced transport problems and unstructured work schedules, while covering a range of tasks usually managed by other staff in health facilities [41, 42]. While some nurses viewed the additional workload as part of their professional duty, others found it exhausting [14, 22]. Handwritten documentation of patient information and its management was reported as a contributing factor to the high workload. In the fixed clinics we observed, consultations were conducted using electronic medical record systems which captured patient data at the point-of-care, and which also helped guide clinical decision-making. As suggested by some of the nurses, a carefully designed system of electronic mobile devices to enter data at point-of-care, would help to reduce workload and is a more efficient way of capturing patient data to the central database.

One key finding from this study was the importance of health providers in facilitating community HIV treatment. This nurse-led community-based model created a comfortable space for patients to reflect on personal problems related to ART and communicate it with their care provider. Although nurses thought that this sometimes led to lack of structure during consultations, findings show that it is crucial for patients to talk to an empathetic and trusted care provider in regard to retention to care. A study conducted in Zambia [36] suggested that when health workers reduced their social distance from patients, patients were more free and open with their care providers. This approach improved patient retention rates by building patients’ confidence and motivating them to remain in care [43–46].

The study revealed that nurses face considerable operational challenges while providing care in the community. Organisations or Ministries of Health planning similar DOMCs led by nurses, need to purposefully plan for the additional transportation costs for staff, the increase in workload, and an efficient system for patient data collection in the field. When identifying DMOC sites patient confidentiality and spaces where private issues can be comfortably discussed, must be taken into consideration. These additional costs must be weighed against the increase in patient retention rates and the decongestion of clinics, which may depend on the availability of resources (including trained human resources) and the drive to control the HIV epidemic.

While this study sheds light on the benefits and challenges of community-level ART delivery, it is acknowledged that response bias may have played a role during patient interviews. More women than men were registered in NCAP which meant that fewer men were interviewed than planned. The reasons for this gender imbalance in accessing NCAP should be explored in future studies. Additionally, this study does not have information on the people who are not accessing NCAP and thus their views of how the model works. Future explorations should seek to compare the different perspectives of this model between those who access it and those who do not.

## Conclusion

The patients interviewed for this study preferred the NCAP approach to the facility-based model of care because it saved money on transport, reduced waiting-times, and gave patients more time to discuss health-related issues during consultations. The decentralisation of HIV care into the community also had a positive impact for fixed health facilities, reducing patient caseload and freeing up capacity, while, providing an incentive for stable patients (who no longer had to wait), and improving patient-provider relationships. However, when considering a DMOC approach, certain factors – including staff transportation and workload, patient privacy, and effective systems for data collection – must be purposefully planned.

## Supplementary information

**Additional file 1.** In-depth interview guide for patients accessing care through the NCAP.

**Additional file 2.** In-depth interview guide for nurses delivering care through the NCAP.

## Data Availability

To protect potential personally identifying information, Lighthouse Trust has not made study data available for the public. Individuals interested in accessing data used in this study can request and obtain the full, anonymised transcripts by emailing Lighthouse Trust at exec_assistant@lighthouse.org.mw.

## Abbreviations

DMOC: Differentiated models of care
ART: Antiretroviral therapy
PLHIV: People living with HIV
NCAP: Nurse-led community ART programme
KCH: Kamuzu Central Hospital
MPC: Martin Preuss Centre
CHS: Community Health Services
ARVs: Antiretroviral drugs
