# Supplementary material for: Patient and nurse perspectives of a nurse-led community-based model of HIV care delivery in Malawi: a qualitative study
Source: BMC Public Health. 2020 May 14;20:685. doi: 10.1186/s12889-020-08721-6 (PMC7227037; doi:10.1186/s12889-020-08721-6)
Supplement: Supplementary file 1 — Additional file 1. In-depth interview guide for patients accessing care through the NCAP. [file 12889_2020_8721_MOESM1_ESM.docx]

**In-depth interview guide for patients accessing care through the NCAP**

Thank you for agreeing to take part in this interview. As discussed in the informed consent, everything you tell me will remain confidential, and there are no right or wrong answers to my questions. You are free to stop the interview at any time and you do not have to answer any questions that make you feel uncomfortable or upset.

**Patients’ perception**

1. Can you tell me a little bit about yourself?

**Prompts**: age, gender, marital status, family background, number of children, education, occupation

1. How did you know about the Nurse-led community ART program?

**Prompts**: source e.g. clinic, radio, friends,

1. How did you take the decision to take part in the NCAP?

**Prompts**: What happened at home that made you come here? Decision taken alone, talked to friends, husband, wife, peers, family setting, asked peers who know the NCAP, etc.

1. What was your experience during the first ART visit at the community?

**Prompts**: reception by the nurse, meeting others, place of meeting, care received

1. What was your experience when you came home after the NCAP care visits?

**Prompts**: reaction of the family, husband, wife; family’s perception and reactions

1. May you describe the kind of care you receive during the nurse-led community ART visits?

**Prompts**: psycho-social support, drugs for opportunistic diseases, tuberculosis, high blood pressure, family planning, etc.

1. Now that you have accessed ARVs through this program what is your general view of it?

**Prompts**: perception of the program, place, nurses’ conduct etc.

1. How would you compare the NCAP visit to the clinic ART visit?

**Prompts:** How different is the NCAP compared to the regular clinic visit? How do the two visits differ in terms of services delivered, distance, waiting time, nurses’ behaviour, etc.?

1. Which one would you recommend to a person on ART, the NCAP program or the ART clinic? Could you explain why?

**Prompts**: Access, distance, time, finances, interaction, etc.

**Benefits of NCAP to the patient**

1. What are the advantages of accessing care through the Nurse-led community ART program?

**Prompts**: time, distance, personal engagements

1. How much time do you usually spend at the support group on your appointment date?

**Prompts**: is it different from different appointment dates? Is it different from time that you spend at the clinic?

1. How do you get to the meeting place?

**Prompts**: how much time do you spend on your way to the support group place? Do you need transport money to get to the meeting place?

1. How many times do you normally access care through NCAP in 3 months?

**Prompts**: missing appointments, planning for the appointments,

1. What might be the reason to miss an appointment at the support group?

**Prompts**: disease, family issues, other occupations, job, no reminder sent, etc.

**Challenges faced by patients**

1. What has been challenging in accessing ART care through the Nurse-led community ART program?

**Prompts**: place, community stigma, interaction with others, interaction with the nurse

1. How do you deal with the challenges?

**Prompts**: friends, family, psycho-social support

1. What would you suggest should be included in the Nurse-led community ART program?

**Prompts**: plays, food, group work that would trigger group dynamics, sending reminder of the appointment, etc.
