# Supplementary material for: Patient and nurse perspectives of a nurse-led community-based model of HIV care delivery in Malawi: a qualitative study
Source: BMC Public Health. 2020 May 14;20:685. doi: 10.1186/s12889-020-08721-6 (PMC7227037; doi:10.1186/s12889-020-08721-6)
Supplement: Supplementary file 2 — Additional file 2. In-depth interview guide for nurses delivering care through the NCAP. [file 12889_2020_8721_MOESM2_ESM.docx]

**In-depth interview guide for nurses delivering care through the NCAP**

Thank you for agreeing to talk to me today. As discussed in the informed consent, everything you tell me will remain confidential, and there are no right or wrong answers to my questions. You are free to stop the interview at any time and you do not have to answer any questions that make you feel uncomfortable or upset.

**Nurses’ perception of the NCAP**

1. What is your role in NCAP service delivery?
2. How long have been providing care through the Nurse-led community ART program?
3. What kind of services do you provide to the patients when they are accessing care on their appointment dates at the support group?

**Prompts**: how does it differ from delivering care in clinic visit?

1. What is the range in number of patients you assist on a given NCAP date through the Nurse-led community ART program?

**Prompts**: maximum number of patients seen in a day, on a busy day or less busy day etc.

1. Do you visit the support groups apart from the ART visit? If so, how is the nurse-patient interaction? Is it different from the ART visit?

**Prompts**: relationship with the patients, support group members, personal relations, etc.

1. How do you interact with patients during the appointment date?

**Prompts**: talking, examining, giving advice, asking questions about life, health, family, well-being, reminding them of the appointments, etc.

1. As a service provider directly involved in this program what do you think about it in general?

**Prompts**: workload, empathy with patient, feeling good about doing good or feeling dissatisfied etc.

**Benefits of the nurse-led ART program**

1. What are the differences between an ART clinic and the Nurse-led ART program for a nurse?

**Prompts**: going to different locations, changing places, seeing different patients, workload, etc.

1. As a provider which mode of HIV care provision do you prefer to provide, and why?

**Prompts**: workload, moving to different locations, time consuming, quality of care, etc.

1. When placing yourself in patients’ place, which mode of HIV care would you recommend for a patient needing ART care and why?

**Prompts**: practical reasons, distance, transport, stigma, quality of care, etc.

1. For you as a provider, what are the advantages of proving care through the Nurse-led community program?

**Prompts**: interaction with the patient, personal relationship, quality of care

**Challenges faced by nurses**

1. What do you find challenging as you are providing care through the Nurse-led community ART program?

**Prompts**: Are they clinic or support group related challenges, personal, institutional

1. How do you deal with challenges?

**Prompts**: Supervision, talking to friends, family support, peer support

1. Are there any improvements that can be made in the Nurse-led community ART program? If yes, in what areas?

**Prompts**: What would you need to feel supported in your work?
